# Poly[[diaquabis(μ_2_-4,4′-bipyridyl)iron(II)] bis{2-[(3-carboxypyridin-2-yl)disulfanyl]nicotinate}]

**DOI:** 10.1107/S1600536811052287

**Published:** 2011-12-10

**Authors:** Jie-Jie Shan, Sheng-Yuan Chai, Yun-Long Feng

**Affiliations:** a Department of Chemistry and Life Science, Zhejiang Normal University, Jinhua 321004, Zhejiang, People’s Republic of China

## Abstract

In the title compound, {[Fe(C_10_H_8_N_2_)_2_(H_2_O)_2_](C_12_H_7_N_2_O_4_S_2_)_2_}_*n*_, synthesized by hydro­thermal reaction, the 4,4′-bipyridyl ligands (one with symmetry 2, one with symmetry 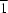) connect Fe^2+^ cations, forming a cationic layer parallel to (001). The coordination of the Fe^2+^ cation (site symmetry 2) is octahedral, with four N atoms from four 4,4′-bipyridyl ligands and O atoms from two *trans* water molecules. Adjacent layers are linked with each other by inter­molecular O—H⋯O hydrogen bonds, forming a three-dimensional supra­molecular structure. Parts of the nicotinic acid derivative are equally disordered over two sets of sites.

## Related literature

For related structures, see: Smith & Sagatys (2003 ▶); Panagiotis *et al.* (2003 ▶); Wang *et al.* (2011 ▶).
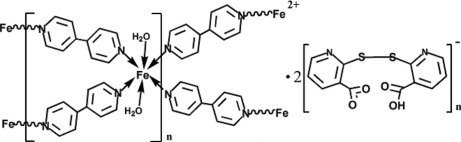


## Experimental

### 

#### Crystal data

[Fe(C_10_H_8_N_2_)_2_(H_2_O)_2_](C_12_H_7_N_2_O_4_S_2_)_2_
*M* *_r_* = 1018.92
Monoclinic, 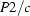

*a* = 11.5161 (2) Å
*b* = 11.6531 (2) Å
*c* = 16.3216 (3) Å
β = 102.403 (1)°
*V* = 2139.21 (7) Å^3^
*Z* = 2
Mo *K*α radiation
μ = 0.62 mm^−1^
*T* = 296 K
0.21 × 0.07 × 0.05 mm

#### Data collection

Bruker APEXII CCD diffractometer
Absorption correction: multi-scan (*SADABS*; Sheldrick, 1996 ▶) *T*_min_ = 0.938, *T*_max_ = 0.957
32774 measured reflections
4930 independent reflections
3286 reflections with *I* > 2σ(*I*)
*R*_int_ = 0.064

#### Refinement

*R*[*F* ^2^ > 2σ(*F* ^2^)] = 0.047
*wR*(*F* ^2^) = 0.126
*S* = 1.05
4930 reflections
389 parameters
127 restraints
H atoms treated by a mixture of independent and constrained refinement
Δρ_max_ = 0.36 e Å^−3^
Δρ_min_ = −0.58 e Å^−3^

### 

Data collection: *APEX2* (Bruker, 2006 ▶); cell refinement: *SAINT* (Bruker, 2006 ▶); data reduction: *SAINT*; program(s) used to solve structure: *SHELXS97* (Sheldrick, 2008 ▶); program(s) used to refine structure: *SHELXL97* (Sheldrick, 2008 ▶); molecular graphics: *DIAMOND* (Brandenburg, 2007 ▶); software used to prepare material for publication: *SHELXL97*.

## Supplementary Material

Crystal structure: contains datablock(s) I, global. DOI: 10.1107/S1600536811052287/rk2316sup1.cif

Structure factors: contains datablock(s) I. DOI: 10.1107/S1600536811052287/rk2316Isup2.hkl

Additional supplementary materials:  crystallographic information; 3D view; checkCIF report

## Figures and Tables

**Table 1:** Hydrogen-bond geometry (Å, °)

*D*—H⋯*A*	*D*—H	H⋯*A*	*D*⋯*A*	*D*—H⋯*A*
O1*W*—H1*WA*⋯O3^i^	0.86 (2)	1.78 (2)	2.634 (3)	172 (3)
O1*W*—H1*WB*⋯O4^ii^	0.83 (2)	1.96 (2)	2.788 (3)	177 (3)
O2—H2⋯O4^iii^	0.86 (2)	1.74 (4)	2.549 (6)	156 (8)
O2′—H2′⋯O4^iii^	0.87 (2)	1.93 (3)	2.776 (10)	164 (9)

Symmetry codes: (i) 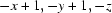; (ii) 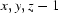; (iii) 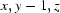.

**Fig. 1.**
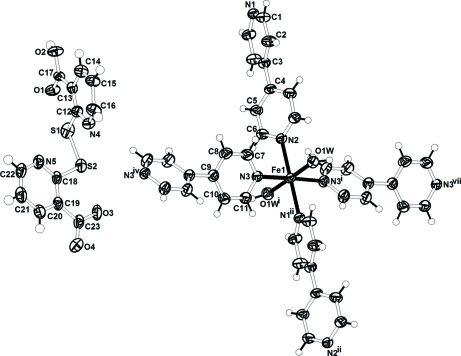
The molecular structure of title compound with the atom numbering scheme. Displacement ellipsoids are drawn at the 30% probability level. H atoms are presented as a small spheres of arbitrary radius. Symmetry codes: (i) -x+1, y, -z-1/2; (ii) x, y+1, z; (iv) -x, y, -z-1/2; (vii) 1+x, y, z.

**Fig. 2.**
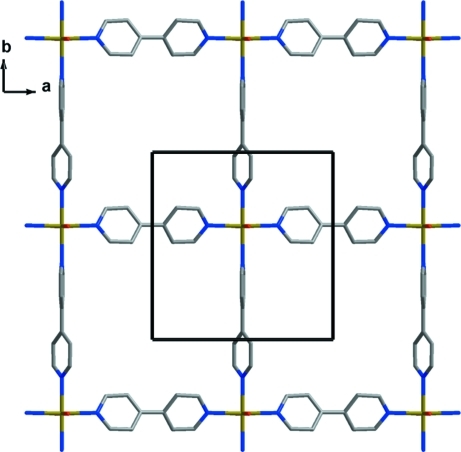
A two-dimensional layered structure (anion molecules are omitted for clarity).

**Fig. 3.**
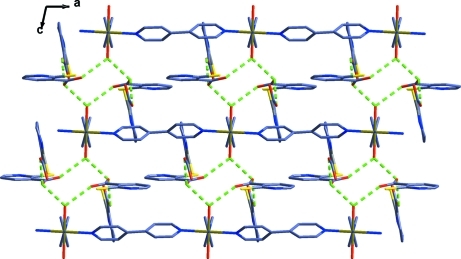
View of the three-dimensional network involving hydrogen bonds.

## supplementary crystallographic information

### Comment

The 2-mercaptopyridine-3-carboxylic acid is an interesting ligand because of its potential versatile coordinate behavior. It may act as a deprotonated ligand through either the carboxylate or the thiolate group, such as 2-mercaptopyridine-3-carboxylate hydrate (Smith *et al.*, 2003) or 2-mercapto-nicotinic acid (Panagiotis *et al.*, 2003). Meanwhile, 2-mercaptopyridine-3-carboxylic acid can produce its derivative, 2-(3-carboxy-pyridine-2-yl disulfanyl)-nicotinic acid under hydrothermal reaction (Wang *et al.*, 2011). As shown in Fig. 1, the Fe atom is six-coordinated by four nitrogen atoms from four *bipy* ligand and two coordinated water molecules in an octahedral configuration. Fe centers are interconnected by neutral *bipy* ligands to form a two-dimensional cationic square-grid layer (Fig. 2). The layers are stacking along the crystallographic *c* axis and the free *L* ligands act as charge-compensating anions (Fig. 3). The intermolecular O–H···O hydrogen bonds between the coordinated water molecules and the uncoordinated carboxylate oxygen atoms play an important role in the formation of the three-dimensional network.

### Experimental

All reagents were purchased commercially and used without further purification. The mixture of 2-mercaptopyridine-3-carboxylic acid (0.0465 g, 0.3 mmol), FeSO_4_×7H_2_O (0.0834 g, 0.3 mmol), *bipy* (0.0468 g, 0.3 mmol) and H_2_O (16 ml) was placed into a 25 ml Teflon-leaned reactor and kept under autogenous pressure at 433 K for 3 days. The mixture was cooled to room temperature at a rate of 5 degrees per hour. The crystals were filtered and washed with water. Then the single crystals suitable for X-ray diffraction were obtained in the mother solution.

### Refinement

The C-bound H-atoms were positioned geometrically and included in the refinement using a riding model with C–H = 0.93Å and *U*_iso_(H) = 1.2*U*_eq_(C). The O-bound H-atoms (water molecule) were located in a difference fourier maps and refined freely with *U*_iso_(H) = 1.2*U*_eq_(O). One of the nicotinic parts is disordered over two occupation sites: N4 C12 C13 C14 C15 C16 C17 O1 O2 and N4' C12' C13' C14' C15' C16' C17' O1' O2' with refined site-occupation factors of 0.5 : 0.5.

### Crystal data

**Table:**

[Fe(C_10_H_8_N_2_)_2_(H_2_O)_2_](C_12_H_7_N_2_O_4_S_2_)_2_	*F*(000) = 1048
*M**_r_* = 1018.92	*D*_x_ = 1.582 Mg m^−^^3^
Monoclinic, *P*2/*c*	Mo *K*α radiation, λ = 0.71073 Å
Hall symbol: -P 2yc	Cell parameters from 3520 reflections
*a* = 11.5161 (2) Å	θ = 1.8–27.6°
*b* = 11.6531 (2) Å	µ = 0.62 mm^−^^1^
*c* = 16.3216 (3) Å	*T* = 296 K
β = 102.403 (1)°	Block, red
*V* = 2139.21 (7) Å^3^	0.21 × 0.07 × 0.05 mm
*Z* = 2	

### Data collection

**Table:**

Bruker APEXII CCD diffractometer	4930 independent reflections
Radiation source: fine-focus sealed tube	3286 reflections with *I* > 2σ(*I*)
graphite	*R*_int_ = 0.064
ω scans	θ_max_ = 27.6°, θ_min_ = 1.8°
Absorption correction: multi-scan (*SADABS*; Sheldrick, 1996)	*h* = −14→14
*T*_min_ = 0.938, *T*_max_ = 0.957	*k* = −14→15
32774 measured reflections	*l* = −21→21

### Refinement

**Table:**

Refinement on *F*^2^	Primary atom site location: structure-invariant direct methods
Least-squares matrix: full	Secondary atom site location: difference Fourier map
*R*[*F*^2^ > 2σ(*F*^2^)] = 0.047	Hydrogen site location: inferred from neighbouring sites
*wR*(*F*^2^) = 0.126	H atoms treated by a mixture of independent and constrained refinement
*S* = 1.05	*w* = 1/[σ^2^(*F*_o_^2^) + (0.0592*P*)^2^ + 0.4305*P*] where *P* = (*F*_o_^2^ + 2*F*_c_^2^)/3
4930 reflections	(Δ/σ)_max_ = 0.001
389 parameters	Δρ_max_ = 0.36 e Å^−^^3^
127 restraints	Δρ_min_ = −0.58 e Å^−^^3^

### Special details

**Table:**

Geometry. All s.u.'s (except the s.u. in the dihedral angle between two l.s. planes) are estimated using the full covariance matrix. The cell s.u.'s are taken into account individually in the estimation of s.u.'s in distances, angles and torsion angles; correlations between s.u.'s in cell parameters are only used when they are defined by crystal symmetry. An approximate (isotropic) treatment of cell s.u.'s is used for estimating s.u.'s involving l.s. planes.
Refinement. Refinement of *F*^2^ against ALL reflections. The weighted *R*-factor *wR* and goodness of fit *S* are based on *F*^2^, conventional *R*-factors *R* are based on *F*, with *F* set to zero for negative *F*^2^. The threshold expression of *F*^2^ > σ(*F*^2^) is used only for calculating *R*-factors(gt) *etc*. and is not relevant to the choice of reflections for refinement. *R*-factors based on *F*^2^ are statistically about twice as large as those based on *F*, and *R*-factors based on ALL data will be even larger.

### Fractional atomic coordinates and isotropic or equivalent isotropic displacement parameters (Å^2^)

**Table:**

	*x*	*y*	*z*	*U*_iso_*/*U*_eq_	Occ. (<1)
Fe1	0.5000	0.60631 (4)	−0.2500	0.02882 (15)	
S1	0.30455 (9)	−0.03861 (10)	0.46412 (6)	0.0812 (3)	
S2	0.33622 (7)	0.13250 (9)	0.46478 (5)	0.0673 (3)	
O3	0.3830 (2)	0.3534 (3)	0.47678 (15)	0.0871 (9)	
O4	0.2747 (2)	0.4971 (3)	0.51206 (17)	0.0838 (8)	
O1W	0.46496 (16)	0.60454 (16)	−0.38083 (11)	0.0384 (4)	
H1WA	0.5140 (18)	0.612 (2)	−0.4132 (14)	0.046*	
H1WB	0.4066 (17)	0.574 (2)	−0.4120 (15)	0.046*	
N1	0.5000	−0.1992 (2)	−0.2500	0.0320 (6)	
N2	0.5000	0.4115 (2)	−0.2500	0.0322 (6)	
N3	0.30520 (16)	0.60959 (17)	−0.25723 (13)	0.0339 (5)	
N5	0.1051 (3)	0.1215 (3)	0.46728 (17)	0.0726 (9)	
C1	0.4545 (2)	−0.1388 (2)	−0.31906 (16)	0.0403 (6)	
H1A	0.4216	−0.1786	−0.3679	0.048*	
C2	0.4538 (2)	−0.0207 (2)	−0.32156 (16)	0.0446 (7)	
H2A	0.4222	0.0170	−0.3716	0.054*	
C3	0.5000	0.0423 (3)	−0.2500	0.0348 (8)	
C4	0.5000	0.1695 (3)	−0.2500	0.0322 (7)	
C5	0.4950 (2)	0.2323 (2)	−0.17883 (16)	0.0404 (6)	
H5A	0.4918	0.1947	−0.1291	0.048*	
C6	0.4948 (2)	0.3499 (2)	−0.18169 (16)	0.0392 (6)	
H6A	0.4908	0.3894	−0.1329	0.047*	
C7	0.2323 (2)	0.5242 (2)	−0.28880 (18)	0.0444 (7)	
H7A	0.2638	0.4626	−0.3129	0.053*	
C8	0.1132 (2)	0.5219 (2)	−0.28784 (18)	0.0450 (7)	
H8A	0.0665	0.4598	−0.3105	0.054*	
C9	0.06347 (19)	0.6128 (2)	−0.25276 (15)	0.0347 (5)	
C10	0.1365 (2)	0.7045 (2)	−0.22368 (16)	0.0400 (6)	
H10A	0.1061	0.7693	−0.2026	0.048*	
C11	0.2552 (2)	0.6987 (2)	−0.22625 (17)	0.0399 (6)	
H11A	0.3035	0.7606	−0.2052	0.048*	
C18	0.1968 (3)	0.1929 (3)	0.47415 (17)	0.0599 (9)	
C19	0.1879 (3)	0.3118 (3)	0.48620 (18)	0.0618 (9)	
C20	0.0773 (3)	0.3543 (4)	0.4883 (2)	0.0778 (11)	
H20A	0.0675	0.4325	0.4960	0.093*	
C21	−0.0196 (3)	0.2817 (5)	0.4791 (3)	0.0884 (13)	
H21A	−0.0951	0.3100	0.4792	0.106*	
C22	−0.0011 (3)	0.1677 (5)	0.4699 (2)	0.0822 (13)	
H22A	−0.0658	0.1186	0.4650	0.099*	
C23	0.2897 (3)	0.3938 (4)	0.4916 (2)	0.0703 (11)	
O1	0.3004 (5)	−0.2480 (5)	0.4844 (3)	0.0697 (17)	0.50
O2	0.2225 (6)	−0.3746 (4)	0.3829 (3)	0.0763 (15)	0.50
H2	0.252 (7)	−0.427 (5)	0.418 (4)	0.092*	0.50
N4	0.2052 (17)	0.026 (3)	0.3020 (18)	0.055 (4)	0.50
C12	0.2344 (3)	−0.0670 (3)	0.3587 (2)	0.0575 (8)	0.50
C13	0.2216 (8)	−0.1758 (10)	0.3477 (6)	0.051 (3)	0.50
C14	0.1735 (11)	−0.2053 (13)	0.2620 (9)	0.072 (3)	0.50
H14A	0.1626	−0.2820	0.2466	0.087*	0.50
C15	0.1438 (13)	−0.1219 (14)	0.2032 (9)	0.071 (4)	0.50
H15A	0.1198	−0.1415	0.1468	0.085*	0.50
C16	0.150 (2)	0.005 (3)	0.230 (2)	0.085 (7)	0.50
H16A	0.1138	0.0622	0.1935	0.102*	0.50
C17	0.2512 (9)	−0.2712 (7)	0.4106 (5)	0.046 (2)	0.50
O1'	0.1141 (7)	−0.3593 (6)	0.3517 (4)	0.123 (2)	0.50
O2'	0.2801 (8)	−0.2910 (9)	0.4351 (7)	0.087 (3)	0.50
H2'	0.292 (8)	−0.353 (5)	0.465 (5)	0.105*	0.50
N4'	0.2197 (17)	0.014 (3)	0.3108 (19)	0.064 (6)	0.50
C12'	0.2344 (3)	−0.0670 (3)	0.3587 (2)	0.0575 (8)	0.50
C13'	0.1886 (9)	−0.1804 (8)	0.3196 (8)	0.054 (3)	0.50
C14'	0.1310 (11)	−0.1877 (13)	0.2374 (10)	0.081 (4)	0.50
H14B	0.1020	−0.2578	0.2144	0.097*	0.50
C15'	0.1163 (15)	−0.0891 (14)	0.1885 (12)	0.091 (5)	0.50
H15B	0.0703	−0.0859	0.1342	0.109*	0.50
C16'	0.1714 (18)	−0.006 (3)	0.2251 (16)	0.062 (4)	0.50
H16B	0.1827	0.0541	0.1898	0.074*	0.50
C17'	0.1925 (7)	−0.2874 (7)	0.3708 (5)	0.073 (2)	0.50

### Atomic displacement parameters (Å^2^)

**Table:**

	*U*^11^	*U*^22^	*U*^33^	*U*^12^	*U*^13^	*U*^23^
Fe1	0.0244 (2)	0.0232 (3)	0.0404 (3)	0.000	0.01043 (18)	0.000
S1	0.0802 (6)	0.0940 (8)	0.0628 (6)	0.0049 (6)	0.0006 (5)	0.0307 (5)
S2	0.0547 (5)	0.0969 (8)	0.0490 (5)	−0.0114 (4)	0.0084 (4)	−0.0028 (4)
O3	0.0699 (16)	0.129 (2)	0.0725 (16)	−0.0343 (16)	0.0374 (13)	−0.0150 (15)
O4	0.0838 (18)	0.079 (2)	0.0859 (19)	−0.0177 (15)	0.0127 (14)	0.0126 (16)
O1W	0.0383 (9)	0.0388 (11)	0.0394 (10)	−0.0043 (8)	0.0108 (7)	−0.0009 (8)
N1	0.0319 (14)	0.0256 (16)	0.0392 (16)	0.000	0.0095 (12)	0.000
N2	0.0324 (14)	0.0258 (17)	0.0400 (16)	0.000	0.0108 (12)	0.000
N3	0.0261 (9)	0.0307 (12)	0.0467 (12)	−0.0017 (9)	0.0119 (8)	−0.0043 (9)
N5	0.0644 (18)	0.104 (3)	0.0502 (16)	−0.0275 (17)	0.0152 (13)	−0.0067 (16)
C1	0.0499 (15)	0.0267 (15)	0.0411 (15)	0.0011 (11)	0.0026 (12)	−0.0038 (11)
C2	0.0613 (17)	0.0296 (15)	0.0385 (15)	0.0019 (12)	0.0009 (12)	0.0020 (12)
C3	0.0365 (18)	0.027 (2)	0.040 (2)	0.000	0.0068 (14)	0.000
C4	0.0347 (17)	0.0203 (19)	0.041 (2)	0.000	0.0070 (14)	0.000
C5	0.0578 (16)	0.0274 (15)	0.0374 (14)	−0.0015 (12)	0.0134 (12)	0.0023 (11)
C6	0.0511 (15)	0.0272 (14)	0.0415 (15)	−0.0001 (12)	0.0147 (12)	−0.0033 (11)
C7	0.0354 (13)	0.0355 (16)	0.0649 (18)	0.0020 (11)	0.0168 (12)	−0.0106 (13)
C8	0.0319 (12)	0.0350 (16)	0.0682 (19)	−0.0046 (11)	0.0111 (12)	−0.0094 (13)
C9	0.0276 (11)	0.0350 (14)	0.0423 (14)	0.0026 (11)	0.0092 (10)	0.0020 (11)
C10	0.0325 (12)	0.0377 (16)	0.0515 (15)	0.0028 (11)	0.0128 (11)	−0.0074 (12)
C11	0.0291 (12)	0.0378 (16)	0.0541 (16)	−0.0038 (11)	0.0115 (11)	−0.0079 (13)
C18	0.0533 (17)	0.094 (3)	0.0345 (15)	−0.0192 (18)	0.0133 (12)	−0.0051 (16)
C19	0.0553 (18)	0.094 (3)	0.0382 (16)	−0.0185 (18)	0.0137 (13)	−0.0071 (17)
C20	0.066 (2)	0.102 (3)	0.070 (2)	−0.008 (2)	0.0246 (18)	−0.014 (2)
C21	0.057 (2)	0.135 (5)	0.077 (3)	−0.010 (3)	0.0231 (18)	−0.009 (3)
C22	0.062 (2)	0.128 (4)	0.059 (2)	−0.039 (3)	0.0182 (17)	−0.010 (2)
C23	0.068 (2)	0.100 (3)	0.0446 (18)	−0.024 (2)	0.0177 (16)	0.001 (2)
O1	0.089 (4)	0.056 (3)	0.053 (3)	−0.007 (3)	−0.010 (3)	−0.002 (3)
O2	0.113 (4)	0.039 (3)	0.066 (3)	−0.003 (3)	−0.003 (3)	0.000 (2)
N4	0.073 (7)	0.044 (6)	0.043 (6)	0.005 (6)	0.000 (6)	0.015 (5)
C12	0.0566 (18)	0.046 (2)	0.072 (2)	0.0075 (15)	0.0172 (15)	0.0074 (17)
C13	0.050 (5)	0.066 (6)	0.036 (5)	0.001 (4)	0.006 (4)	0.000 (4)
C14	0.078 (6)	0.073 (6)	0.061 (6)	0.003 (5)	0.003 (5)	−0.003 (5)
C15	0.088 (7)	0.069 (8)	0.048 (5)	0.003 (6)	−0.003 (5)	−0.012 (5)
C16	0.099 (11)	0.084 (9)	0.067 (8)	0.010 (8)	0.008 (7)	−0.012 (6)
C17	0.041 (4)	0.053 (5)	0.041 (4)	−0.008 (4)	−0.001 (3)	−0.017 (4)
O1'	0.149 (6)	0.102 (5)	0.116 (5)	−0.055 (5)	0.020 (4)	−0.003 (4)
O2'	0.064 (5)	0.088 (6)	0.108 (6)	−0.002 (4)	0.015 (4)	0.037 (5)
N4'	0.070 (6)	0.054 (9)	0.054 (8)	0.012 (5)	−0.014 (4)	0.005 (6)
C12'	0.0566 (18)	0.046 (2)	0.072 (2)	0.0075 (15)	0.0172 (15)	0.0074 (17)
C13'	0.055 (5)	0.043 (4)	0.063 (7)	−0.004 (4)	0.013 (5)	0.002 (5)
C14'	0.085 (7)	0.063 (7)	0.093 (8)	−0.025 (6)	0.015 (6)	−0.025 (6)
C15'	0.099 (8)	0.077 (8)	0.091 (8)	−0.005 (6)	0.006 (6)	−0.011 (6)
C16'	0.063 (6)	0.073 (8)	0.046 (6)	0.002 (6)	0.003 (5)	−0.001 (5)
C17'	0.074 (5)	0.077 (6)	0.069 (5)	−0.015 (5)	0.020 (4)	−0.020 (4)

### Geometric parameters (Å, °)

**Table:**

Fe1—O1W^i^	2.0863 (17)	C9—C10	1.379 (3)
Fe1—O1W	2.0863 (17)	C9—C9^iv^	1.484 (4)
Fe1—N3	2.2213 (18)	C10—C11	1.378 (3)
Fe1—N3^i^	2.2213 (18)	C10—H10A	0.9300
Fe1—N1^ii^	2.267 (3)	C11—H11A	0.9300
Fe1—N2	2.270 (3)	C18—C19	1.406 (5)
S1—C12	1.768 (4)	C19—C20	1.374 (5)
S1—S2	2.0266 (16)	C19—C23	1.500 (5)
S2—C18	1.789 (3)	C20—C21	1.382 (5)
O3—C23	1.243 (4)	C20—H20A	0.9300
O4—C23	1.272 (5)	C21—C22	1.359 (6)
O1W—H1WA	0.857 (16)	C21—H21A	0.9300
O1W—H1WB	0.828 (16)	C22—H22A	0.9300
N1—C1^i^	1.337 (3)	O1—C17	1.246 (8)
N1—C1	1.337 (3)	O2—C17	1.304 (8)
N1—Fe1^iii^	2.267 (3)	O2—H2	0.86 (2)
N2—C6	1.338 (3)	N4—C16	1.24 (4)
N2—C6^i^	1.338 (3)	N4—C12	1.42 (3)
N3—C7	1.332 (3)	C12—C13	1.284 (12)
N3—C11	1.338 (3)	C13—C14	1.431 (15)
N5—C18	1.330 (4)	C13—C17	1.503 (9)
N5—C22	1.346 (5)	C14—C15	1.36 (2)
C1—C2	1.377 (4)	C14—H14A	0.9300
C1—H1A	0.9300	C15—C16	1.54 (4)
C2—C3	1.385 (3)	C15—H15A	0.9300
C2—H2A	0.9300	C16—H16A	0.9300
C3—C2^i^	1.385 (3)	O1'—C17'	1.222 (8)
C3—C4	1.483 (5)	O2'—C17'	1.291 (8)
C4—C5^i^	1.384 (3)	O2'—H2'	0.87 (2)
C4—C5	1.384 (3)	N4'—C16'	1.41 (4)
C5—C6	1.371 (4)	C13'—C14'	1.367 (17)
C5—H5A	0.9300	C13'—C17'	1.496 (9)
C6—H6A	0.9300	C14'—C15'	1.39 (2)
C7—C8	1.375 (3)	C14'—H14B	0.9300
C7—H7A	0.9300	C15'—C16'	1.24 (3)
C8—C9	1.386 (4)	C15'—H15B	0.9300
C8—H8A	0.9300	C16'—H16B	0.9300
			
O1W^i^—Fe1—O1W	178.87 (10)	C10—C9—C9^iv^	121.02 (17)
O1W^i^—Fe1—N3	91.47 (7)	C8—C9—C9^iv^	121.68 (18)
O1W—Fe1—N3	88.55 (7)	C11—C10—C9	119.2 (2)
O1W^i^—Fe1—N3^i^	88.55 (7)	C11—C10—H10A	120.4
O1W—Fe1—N3^i^	91.47 (7)	C9—C10—H10A	120.4
N3—Fe1—N3^i^	178.03 (11)	N3—C11—C10	124.0 (2)
O1W^i^—Fe1—N1^ii^	90.57 (5)	N3—C11—H11A	118.0
O1W—Fe1—N1^ii^	90.57 (5)	C10—C11—H11A	118.0
N3—Fe1—N1^ii^	89.01 (5)	N5—C18—C19	123.2 (3)
N3^i^—Fe1—N1^ii^	89.01 (5)	N5—C18—S2	117.2 (3)
O1W^i^—Fe1—N2	89.43 (5)	C19—C18—S2	119.5 (2)
O1W—Fe1—N2	89.43 (5)	C20—C19—C18	117.0 (3)
N3—Fe1—N2	90.99 (5)	C20—C19—C23	119.1 (4)
N3^i^—Fe1—N2	90.99 (5)	C18—C19—C23	123.8 (3)
N1^ii^—Fe1—N2	180.0	C19—C20—C21	120.5 (4)
C12—S1—S2	103.51 (13)	C19—C20—H20A	119.7
C18—S2—S1	102.92 (13)	C21—C20—H20A	119.7
Fe1—O1W—H1WA	128.5 (17)	C22—C21—C20	117.9 (4)
Fe1—O1W—H1WB	125.8 (18)	C22—C21—H21A	121.1
H1WA—O1W—H1WB	103 (2)	C20—C21—H21A	121.1
C1^i^—N1—C1	116.5 (3)	N5—C22—C21	124.1 (4)
C1^i^—N1—Fe1^iii^	121.77 (15)	N5—C22—H22A	117.9
C1—N1—Fe1^iii^	121.77 (15)	C21—C22—H22A	117.9
C6—N2—C6^i^	115.1 (3)	O3—C23—O4	125.5 (3)
C6—N2—Fe1	122.47 (15)	O3—C23—C19	116.4 (4)
C6^i^—N2—Fe1	122.47 (15)	O4—C23—C19	118.1 (3)
C7—N3—C11	116.1 (2)	C17—O2—H2	113 (6)
C7—N3—Fe1	123.58 (16)	C16—N4—C12	118 (3)
C11—N3—Fe1	120.31 (16)	C13—C12—N4	131.3 (14)
C18—N5—C22	117.2 (4)	C13—C12—S1	109.6 (5)
N1—C1—C2	123.4 (2)	N4—C12—S1	119.0 (13)
N1—C1—H1A	118.3	C12—C13—C14	112.5 (9)
C2—C1—H1A	118.3	C12—C13—C17	129.2 (8)
C1—C2—C3	120.4 (3)	C14—C13—C17	118.2 (10)
C1—C2—H2A	119.8	C15—C14—C13	120.4 (14)
C3—C2—H2A	119.8	C15—C14—H14A	119.8
C2—C3—C2^i^	116.0 (3)	C13—C14—H14A	119.8
C2—C3—C4	122.01 (17)	C14—C15—C16	119.9 (16)
C2^i^—C3—C4	122.01 (17)	C14—C15—H15A	120.0
C5^i^—C4—C5	116.2 (3)	C16—C15—H15A	120.0
C5^i^—C4—C3	121.90 (16)	N4—C16—C15	117 (3)
C5—C4—C3	121.90 (16)	N4—C16—H16A	121.7
C6—C5—C4	120.0 (2)	C15—C16—H16A	121.7
C6—C5—H5A	120.0	O1—C17—O2	124.4 (8)
C4—C5—H5A	120.0	O1—C17—C13	119.3 (7)
N2—C6—C5	124.4 (2)	O2—C17—C13	116.3 (7)
N2—C6—H6A	117.8	C17'—O2'—H2'	119 (7)
C5—C6—H6A	117.8	C14'—C13'—C17'	116.5 (11)
N3—C7—C8	123.9 (2)	C13'—C14'—C15'	119.1 (13)
N3—C7—H7A	118.0	C16'—C15'—C14'	112.6 (18)
C8—C7—H7A	118.0	C15'—C16'—N4'	131 (3)
C7—C8—C9	119.4 (2)	O1'—C17'—O2'	126.2 (9)
C7—C8—H8A	120.3	O1'—C17'—C13'	119.8 (8)
C9—C8—H8A	120.3	O2'—C17'—C13'	113.9 (9)
C10—C9—C8	117.3 (2)		
			
C12—S1—S2—C18	82.35 (14)	C9—C10—C11—N3	1.5 (4)
O1W^i^—Fe1—N2—C6	13.85 (14)	C22—N5—C18—C19	1.9 (4)
O1W—Fe1—N2—C6	−166.15 (14)	C22—N5—C18—S2	−176.6 (2)
N3—Fe1—N2—C6	−77.61 (14)	S1—S2—C18—N5	−8.6 (2)
N3^i^—Fe1—N2—C6	102.39 (14)	S1—S2—C18—C19	172.9 (2)
O1W^i^—Fe1—N2—C6^i^	−166.15 (14)	N5—C18—C19—C20	−1.9 (5)
O1W—Fe1—N2—C6^i^	13.85 (14)	S2—C18—C19—C20	176.5 (2)
N3—Fe1—N2—C6^i^	102.39 (14)	N5—C18—C19—C23	−178.2 (3)
N3^i^—Fe1—N2—C6^i^	−77.61 (14)	S2—C18—C19—C23	0.2 (4)
O1W^i^—Fe1—N3—C7	−114.2 (2)	C18—C19—C20—C21	0.1 (5)
O1W—Fe1—N3—C7	64.6 (2)	C23—C19—C20—C21	176.6 (3)
N1^ii^—Fe1—N3—C7	155.2 (2)	C19—C20—C21—C22	1.5 (6)
N2—Fe1—N3—C7	−24.8 (2)	C18—N5—C22—C21	−0.1 (5)
O1W^i^—Fe1—N3—C11	63.3 (2)	C20—C21—C22—N5	−1.6 (6)
O1W—Fe1—N3—C11	−117.8 (2)	C20—C19—C23—O3	−169.5 (3)
N1^ii^—Fe1—N3—C11	−27.23 (19)	C18—C19—C23—O3	6.8 (5)
N2—Fe1—N3—C11	152.77 (19)	C20—C19—C23—O4	11.6 (5)
C1^i^—N1—C1—C2	0.54 (19)	C18—C19—C23—O4	−172.2 (3)
Fe1^iii^—N1—C1—C2	−179.46 (19)	C16—N4—C12—C13	6.0 (17)
N1—C1—C2—C3	−1.1 (4)	C16—N4—C12—S1	−175.9 (13)
C1—C2—C3—C2^i^	0.50 (18)	S2—S1—C12—C13	172.7 (5)
C1—C2—C3—C4	−179.50 (18)	S2—S1—C12—N4	−5.8 (7)
C2—C3—C4—C5^i^	−28.97 (19)	N4—C12—C13—C14	2.8 (12)
C2^i^—C3—C4—C5^i^	151.03 (19)	S1—C12—C13—C14	−175.5 (7)
C2—C3—C4—C5	151.03 (19)	N4—C12—C13—C17	−178.2 (12)
C2^i^—C3—C4—C5	−28.97 (19)	S1—C12—C13—C17	3.5 (12)
C5^i^—C4—C5—C6	0.22 (17)	C12—C13—C14—C15	−1.8 (16)
C3—C4—C5—C6	−179.78 (17)	C17—C13—C14—C15	179.0 (12)
C6^i^—N2—C6—C5	0.24 (19)	C13—C14—C15—C16	−6(2)
Fe1—N2—C6—C5	−179.76 (19)	C12—N4—C16—C15	−14 (2)
C4—C5—C6—N2	−0.5 (4)	C14—C15—C16—N4	15 (3)
C11—N3—C7—C8	−2.7 (4)	C12—C13—C17—O1	−3.7 (16)
Fe1—N3—C7—C8	175.0 (2)	C14—C13—C17—O1	175.3 (10)
N3—C7—C8—C9	0.4 (4)	C12—C13—C17—O2	176.2 (9)
C7—C8—C9—C10	2.9 (4)	C14—C13—C17—O2	−4.7 (14)
C7—C8—C9—C9^iv^	−177.7 (3)	C17'—C13'—C14'—C15'	175.0 (12)
C8—C9—C10—C11	−3.7 (4)	C13'—C14'—C15'—C16'	8(2)
C9^iv^—C9—C10—C11	176.9 (3)	C14'—C15'—C16'—N4'	−18 (3)
C7—N3—C11—C10	1.7 (4)	C14'—C13'—C17'—O1'	−27.1 (14)
Fe1—N3—C11—C10	−176.0 (2)	C14'—C13'—C17'—O2'	155.7 (11)

Symmetry codes: (i) −*x*+1, *y*, −*z*−1/2; (ii) *x*, *y*+1, *z*; (iii) *x*, *y*−1, *z*; (iv) −*x*, *y*, −*z*−1/2.

### Hydrogen-bond geometry (Å, °)

**Table:**

*D*—H···*A*	*D*—H	H···*A*	*D*···*A*	*D*—H···*A*
O1W—H1WA···O3^v^	0.86 (2)	1.78 (2)	2.634 (3)	172 (3)
O1W—H1WB···O4^vi^	0.83 (2)	1.96 (2)	2.788 (3)	177 (3)
O2—H2···O4^iii^	0.86 (2)	1.74 (4)	2.549 (6)	156 (8)
O2'—H2'···O4^iii^	0.87 (2)	1.93 (3)	2.776 (10)	164 (9)

Symmetry codes: (v) −*x*+1, −*y*+1, −*z*; (vi) *x*, *y*, *z*−1; (iii) *x*, *y*−1, *z*.
